# Common Molecular Alterations in Canine Oligodendroglioma and Human Malignant Gliomas and Potential Novel Therapeutic Targets

**DOI:** 10.3389/fonc.2019.00780

**Published:** 2019-08-14

**Authors:** Dana Mitchell, Sreenivasulu Chintala, Kaleigh Fetcko, Mario Henriquez, Brij N. Tewari, Atique Ahmed, R. Timothy Bentley, Mahua Dey

**Affiliations:** ^1^Department of Neurosurgery, Simon Cancer Center, Indiana University School of Medicine, Indianapolis, IN, United States; ^2^Department of Neurological Surgery, Northwestern University, Chicago, IL, United States; ^3^Department of Veterinary Clinical Sciences, Purdue University Center for Cancer Research, Purdue University, West Lafayette, IN, United States

**Keywords:** canine glioma, glioblastoma, molecular therapeutic targets, malignant glioma, anaplastic oligodendroglioma

## Abstract

Spontaneous canine (*Canis lupus*) oligodendroglioma (ODG) holds tremendous potential as an immunocompetent large animal model of human malignant gliomas (MG). However, the feasibility of utilizing this model in pre-clinical studies depends on a thorough understanding of the similarities and differences of the molecular pathways associated with gliomas between the two species. We have previously shown that canine ODG has an immune landscape and expression pattern of commonly described oncogenes similar to that of human MG. In the current study, we performed a comprehensive analysis of canine ODG RNAseq data from 4 dogs with ODG and 2 normal controls to identify highly dysregulated genes in canine tumors. We then evaluated the expression of these genes in human MG using Xena Browser, a publicly available database. STRING-database inquiry was used in order to determine the suggested protein associations of these differentially expressed genes as well as the dysregulated pathways commonly enriched by the protein products of these genes in both canine ODG and human MG. Our results revealed that 3,712 (23%) of the 15,895 differentially expressed genes demonstrated significant up- or downregulation (log2-fold change > 2.0). Of the 3,712 altered genes, ~50% were upregulated (*n* = 1858) and ~50% were downregulated (*n* = 1854). Most of these genes were also found to have altered expression in human MG. Protein association and pathway analysis revealed common pathways enriched by members of the up- and downregulated gene categories in both species. In summary, we demonstrate that a similar pattern of gene dysregulation characterizes both human MG and canine ODG and provide additional support for the use of the canine model in order to therapeutically target these common genes. The results of such therapeutic targeting in the canine model can serve to more accurately predict the efficacy of anti-glioma therapies in human patients.

## Introduction

Malignant gliomas (MG) are central nervous system tumors of glial origin characterized by molecular heterogeneity and a poor prognosis. Despite significant advances in the field, the 5-year survival rate for patients with glioblastoma and IDH-wild type anaplastic astrocytoma is still <10% (1). Recent studies using pre-clinical mouse models have led to a better understanding of glioma pathogenesis; however, gaps still remain in the translation of these studies into clinical benefit. One of the factors contributing to this lack of translational benefit is the need for a pre-clinical glioma model capable of more accurately predicting the efficacy of novel anti-glioma therapies in human patients (2). Recent studies have shown that the spontaneous, immunocompetent canine model of glioma may be a solution to this problem given its anatomical, physiological, and molecular similarity to human glioma (2–4). In addition to their biological similarities, human and canine gliomas have demonstrated similar therapeutic responses to chemotherapy, conventional radiotherapy, and stereotactic radiosurgery (3, 5).

Dogs have been shown to spontaneously develop brain tumors exhibiting histopathological, immunological, molecular, and clinical characteristics similar to those observed in human glioma (6). Unlike murine models, canine gliomas, which originate spontaneously in immunocompetent and genetically diverse hosts, will allow for a better understanding of the effect of the host's physiology and immune system on the therapeutic response. Thus, the canine glioma model will be able to more reliably predict the success of anti-glioma therapies in human patients. In order to integrate the canine glioma model into pre-clinical testing and identify novel common therapeutic targets, comprehensive genomic studies of canine brain tumors are required. Several studies evaluating molecular signaling pathways in canine glioma have demonstrated that the expression pattern of key signaling pathway proteins, MDM2, TP53, PTEN, P21, AKT, RB1, mTOR, and MAPK is similar to that seen in human disease (4, 5). We recently demonstrated that, like human MGs, canine ODGs also exhibit dysregulation of PI3K, RAS, PIK3CB, TP53, SOX2, DNMT1, CDK4, CCNB1, ERBB2, OLIG2, and PAK1 (4).

In order to identify common molecular aberrations characterizing gliomas in these two species, which can be used for therapeutic targeting, we comprehensively analyzed canine ODG RNA sequencing data to identify genes that are highly dysregulated. We then evaluated their expression in various grades of human MG and confirmed the RNAseq data at the protein level. In addition, we analyzed the suggested protein associations and KEGG pathways commonly enriched by the associated proteins in both human and canine glioma. In this study, we show that the genes differentially expressed in canine ODG, exhibit similar expression patterns in human MG and that these differentially expressed genes are involved in novel, common pathways that can be targeted therapeutically.

## Materials and Methods

### Analysis of RNAseq Data

RNA sequencing (RNAseq) was performed with two canine normal brains and four canine oligodendroglioma (ODG) samples as previously described (4). RNAseq data is available through NCBI BioProject ID PRJNA557484. A comparison of gene expression in canine glioma and healthy brain tissue was performed using package edge R of R (7). Using the same RNAseq dataset, to encompass larger pool of differentially expressed genes, in this study we analyzed differentially expressed genes using a threshold of fold change (FC) > 2 only. Using this strategy, a total of 3,712 genes were identified as differentially expressed, and from these 3,712 genes, we identified the top 10 significantly upregulated and downregulated genes, as well as the top 10 upregulated cluster differentiation (CD) genes in tumors compared to normal brain.

### Quantitative RT-PCR

Total RNA was isolated from two canine normal brains and three of the four residual tumor tissues using RNeasy Kit (Qiagen,) as per manufacturer's instructions. Complementary DNA (cDNA) was synthesized using the iScript cDNA synthesis kit (Bio-Rad). Canine specific primers were designed using the Primer3 for the selected genes to validate gene expression by quantitative RT-PCR as described earlier (8). RT-PCR blots were analyzed using ImageJ to quantify the intensity of the bands using the band intensity peaks tool and determined the average gene expression in tumor and normal tissue (9). If two bands were present, both bands were used for ImageJ analysis as these were suspected to be isoforms. RT-PCR data full length gel images are provided in Figure S1. Graphs demonstrate the average expression in the tumor samples (*n* = 3) based on Image J quantification analysis. qPCR with three technical replicates using two normal brain and three canine brain samples was performed to confirm the expression of RRM2, DTL, IRX5, CD1A6, CD93, CD163, and CD36. Beta-actin was used as a reference gene for normalization. Primer sequences used to confirm the expression of selected genes by qPCR are reported in the Supplemental Data.

### Western Blot Analysis

Confirmation of the overexpression of KIF11 and UBE2C proteins in canine ODGs was performed by western blot analysis. Frozen tissues from canine tumor and normal brains were used to extract the protein by homogenization in lysis buffer. Equal quantities of protein (40 μg) were separated by electrophoresis using the 4–20% gradient Mini-Protean TGX Stain free gels (Bio-Rad) and transferred to PVDF membrane using the Trans-Blot Turbo Transfer System (Bio-Rad). Blots were blocked with the blocking agent (ECL Prime, GE Health Care) and probed with primary antibody anti-KIF11 (Sigma Aldrich) at 1:500 dilution, anti-UBE2C (Sigma Aldrich), at 1:500 dilution for 1 h at room temperature. After washing, respective HRP conjugated secondary antibodies were used at 1:5,000 dilution. Blots were developed using the ECL Prime Western Blot Detection Reagent (GE Health Care) and Chemi Doc Touch Imaging System (Bio-Rad) was used to detect the expression of KIF11 and UBE2C protein levels. Beta actin was used as the loading control.

### Immunohistochemical Analysis

Formalin fixed paraffin embedded sections of canine tumor and normal brain were used to determine the expression of KIF11 and UBE2C by immunohistochemistry (IHC) as per the protocol described by Cell Signaling Technology IHC protocol-paraffin for SignalStain Boost Detection Reagent. After deparaffinization/rehydration of sections, antigen retrieval was done with 10 mM sodium citrate buffer pH 6.0 maintaining sub-boiling temperature for 10 min using the microwave, and slides were allowed to cool on the bench for 30 min. Sections were subjected to blocking for 1 h with blocking solution, followed by primary antibody anti-KIF11 (Sigma Aldrich), and anti-UBE2C (Sigma Aldrich) incubation overnight at 4°C in a humidified chamber. The primary antibody was removed by washing the sections with wash buffer three times for 5 min each. Sections were covered with respective HRP conjugated secondary antibody for 1 h at room temperature in a humidified chamber. Sections were again washed three times with wash buffer for 5 min each and stained with DAB substrate. Counterstaining was performed with H & E stain. Sections were mounted with cover slips using the Cytoseal mounting medium. After overnight drying the sections, photomicrographs were captured using the EVOS FL microscope (ThermoFisher Scientific).

### Gene Expression Analysis

Genomic expression data for the selected canine ODG genes (Supplemental Data), in human GBM, ODG, OA, AA patients were obtained from Xena Browser using the TCGA database (https://xenabrowser.net) (10). Expression levels of these genes in human GBM, ODG, OA, and AA were compared to their expression in normal brain using the GTEX dataset-primary site: brain, which was also available through (https://xenabrowser.net). The Human Protein Atlas (https://www.proteinatlas.org/pathology) was used to determine the expression of cell cycle regulatory proteins KIF11 and UBE2C in human glioma (11).

### Protein Association Analysis

String Database (https://string-db.org/) was used in order to determine the predicted protein associations of the selected gene products in both humans and canines. For the top 10 up- and down-regulated and top CD genes, the “max number of interactions to show” was set to “no more than 10 interactors.” For all predicted protein association analyses, a minimum required interaction score of 0.400 (medium confidence) was used, with the exception of the interaction map of the top 10 upregulated genes in canines, which could only be performed using a minimum required interaction score of 0.150 (low confidence). For the top 10 up- and down-regulated and CD genes, predicted protein association similarity was calculated by evaluating the number of canine associated proteins also reported in human data and dividing that number by the total number of suggested associated proteins reported in canines. For the top 100 up- and down-regulated genes, the percent similarity was calculated by assessing the number of proteins demonstrating similar interactions and dividing that number by the total number of proteins reported in the canine data.

### Pathway Enrichment Analysis

Pathway enrichment analysis was performed using the STRING database to determine the KEGG pathways reported to be enriched by the associated proteins of the top 10 up- and down-regulated and top 10 CD genes in both human and canine. The percent similarity of KEGG pathway enrichment was calculated by assessing the number of canine pathways that are reported in human database and dividing that number by the total number of canine pathways reported. A false discovery rate of <0.5 was set as the cutoff. KEGG and Biological process GO pathway analysis was also performed for the top 100 up- and downregulated genes. Biological process GO pathways reported in canine were evaluated in human data. Pathways reported with a FDR <0.02 in humans and FDR <0.05 in canines were included.

### Statistical Analysis

Statistical analysis was performed using the GraphPad Prism (GraphPad Software, San Diego, CA). Unpaired *t*-test analysis was performed to determine statistical significance. Specific gene expression of each group of GBM, ODG, OA, and AA was compared with non-tumor (NT). Statistical significance was defined as a *p*-value < 0.05.

## Results

### Overall Gene Expression Landscape of Canine Oligodendroglioma

**Figure 1:**
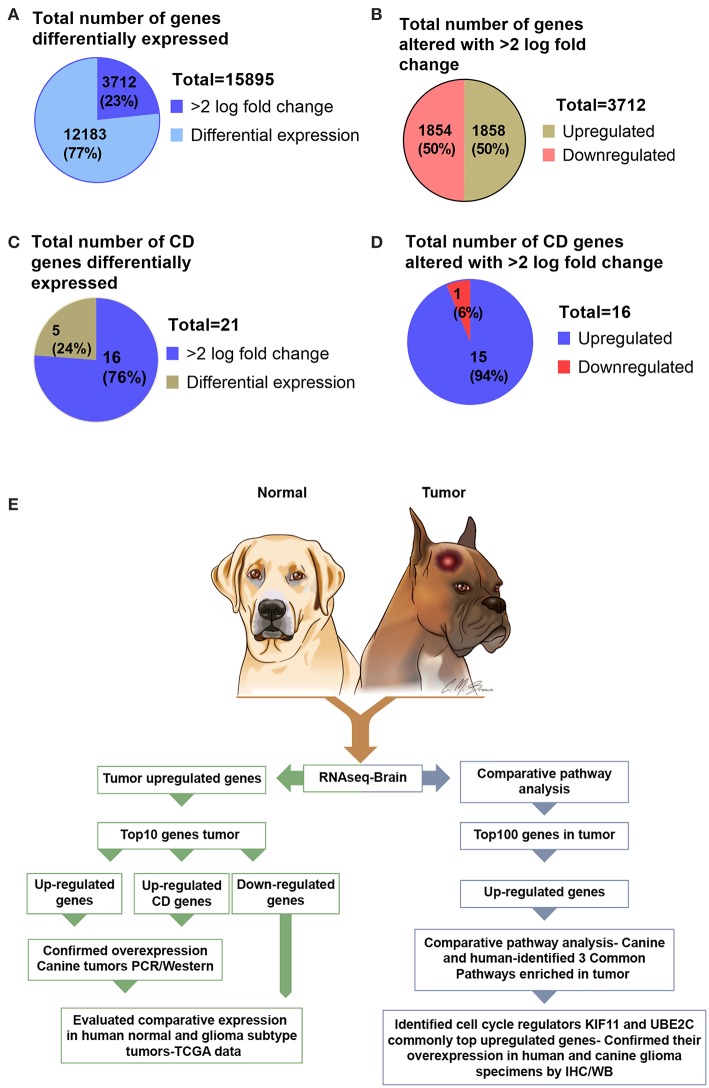
Graphical presentation of the total number of genes differentially expressed in canine ODG and selection strategy of genes for further evaluation in both human and canine glioma. **(A)** Number of differentially expressed genes (*n* = 15,895) and the percentage of genes significantly altered with >2.0 log fold change. **(B)** Percentage of differentially expressed genes that are upregulated and downregulated genes in canine ODG. **(C)** Total number and percentage of CD genes differentially expressed in canine ODG (*n* = 21). **(D)** Percentage of CD genes upregulated and downregulated in canine ODG. **(E)** Gene selection strategy and study summary.

Analysis of RNA sequencing data using FC >2 revealed a total of 15,895 differentially expressed genes in canine ODG. 3,712 (23%) of these differentially expressed genes were found to be up- or down-regulated with log 2-fold change >2.0 in canine tumors compared to normal brains. Approximately 50% (1,858) of these genes were up-regulated and 50% (1,854) were down-regulated (Figures 1A,B). Given the significant role of cluster differentiation (CD) genes in immune modulation, and the lack of studies evaluating the expression of these genes in canine glioma, we further expanded our analysis to determine the CD genes differentially expressed in canine ODG. Our results demonstrated that 21 CD genes are differentially expressed in canine ODG compared to normal brains and among these 21 genes, 16 were found to be significantly altered with log2-fold change >2.0 (Figure 1C). Interestingly, 15 of 16 differentially expressed genes were up-regulated and only one (CD1A) was down-regulated (Figure 1D). Figure 1E summarizes our gene selection and further analysis strategy.

### The Expression Pattern of the Highly Upregulated Genes Identified in Canine ODG Is Similar to Human ODG and GBM

**Figure 2:**
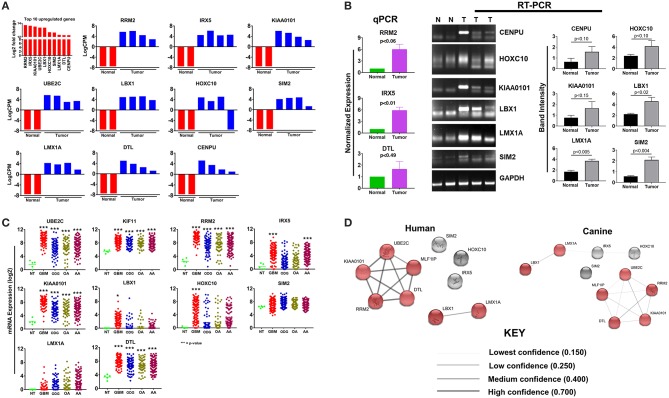
Top 10 upregulated genes in canine oligodendroglioma. **(A)** Expression of the top 10 upregulated genes in canine glioma cases based on RNAseq data. **(B)** qPCR/RT-PCR analysis confirming the upregulation of the top 10 upregulated genes in canine ODG (9 genes; 10th gene by IHC/WB). **Figure 9** compared to normal brain **(C)** expression of the top 10 upregulated genes in human glioma subtypes: Glioblastoma (GBM), Oligodendroglioma (ODG), Oligoastrocytoma (OA), and Anaplastic Astrocytoma (AA), based on data from Xena Browser (TCGA: low grade glioma and glioblastoma). CENPU expression was not available in human data and was, therefore, excluded from this dataset (*p ≤ 0.05, **p ≤ 0.01, ***p ≤ 0.001) **(D)** comparison of the suggested protein associations between the protein products of the top 10 upregulated genes between humans and canines. Data was based on reported associations from the STRING database (functional protein association network version-10-5). Medium confidence (0.400) was used for human patients, whereas low confidence (0.150) was required for the canine data due to lack of supporting data. Red nodes indicate proteins involved in similar interactions in humans and canines.

The top 10 upregulated genes in canine ODG demonstrated a 13.7–12.3 log2-fold increase in the mean expression level (*p*-value of 6.15E-21–1.12E-06; Figure 2A). In order to capture inter-animal tumor heterogeneity, we verified the individual top 10 upregulated gene expression levels in each animal separately. Except for HOXC10, which was down-regulated in one of the canine glioma cases, all other genes were overexpressed in all canine glioma cases. RNAseq data was validated by quantitative RT-PCR (qRT-PCR; Figure 2B). qPCR was used to confirm the expression of RRM2 (*p* = 0.06), IRX5 (*p* = 0.01), and DTL (*p* = 0.49) and RT-PCR data demonstrated significant upregulation of SIM2 (*p* = 0.0047), LMX1A (*p* = 0.0055), and LBX1 (*p* = 0.0225), but not KIAA0101 (*p* = 0.1557), HOXC10 (*p* = 0.1076), or CENPU (*p* = 0.1027). The lack of significant upregulation demonstrated by the RT-PCR data is likely attributed to the small tumor sample size and tumor heterogeneity, which is demonstrated by the variable expression of these genes as shown in Figure 2A. This finding further supports the utility of canine ODG as a model for human glioma, which is known for its vast heterogeneity. Western blot analysis was performed for UBE2C and KIF11 (**Figure 9**). KIF11 bands were observed at 120 and 60 kDa as reported in the literature and are believed to be due to splice variants of the KIF11 gene (12). The observed 60 kDa band in canine brain samples may be a canine-specific variant. The expected band size for UBE2C is ~20 kDa, however, we observed bands at 75, 47, and 28 kDa for UBE2C. We believe that the 75 kDa band may be an isoform or the result of post-translational modification that needs to be validated with future experiments. To further confirm the overexpression of UBE2C and KIF11, immunohistochemistry was performed using antibodies previously optimized in the literature (13, 14).

Utilizing the human genomic database, we observed significant overexpression of 80% (7 of the 9 available) of the top 10 up-regulated canine ODG genes in human ODG and 90% (8 of the 9 available) in human GBM (Figure 2C). The genes *RRM2, KIAA0101, UBE2C, LMX1A, DTL*, and *LBX1* demonstrated significant overexpression in all human glioma subtypes (GBM, ODG, OA, AA) with a *p*-value ≤ 0.0001. *IRX5* was found to be significantly overexpressed in human GBM and AA (*p* ≤ 0.0001) and human OA (*p* ≤ 0.04), but not in human ODG. Additionally*, HOXC10* and *SIM2* demonstrated significant overexpression in human GBM (*p* ≤ 0.0001) and human ODG (*p* ≤ 0.04), respectively, but not in the other human glioma subtypes. Data on *CENPU* gene expression was not available in the human database, and was therefore not included in the human dataset.

**Figure 3:**
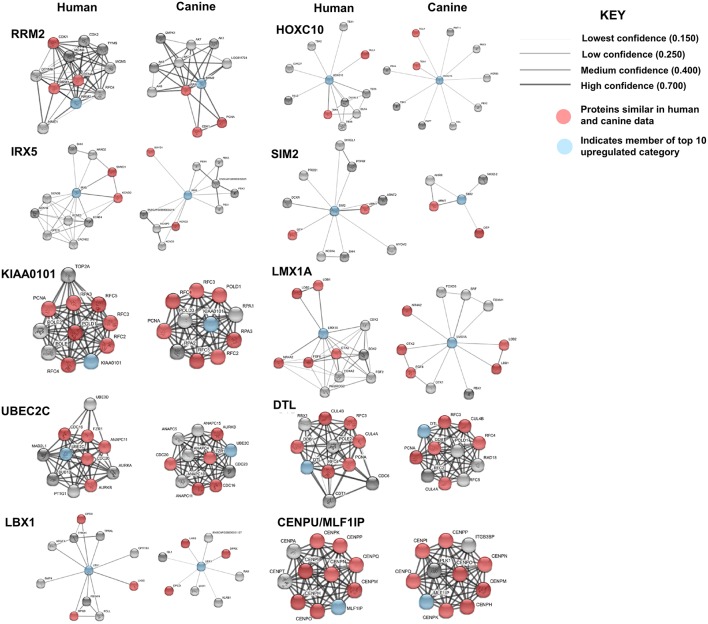
Comparison of suggested protein associations of top 10 upregulated genes in humans and canines. Data was collected using the STRING database (functional protein association network version-10-5) at medium confidence (0.400). Increasing confidence is demonstrated by increased thickness and darkness of edges (intersecting lines). Red notes indicate proteins involved in similar associations in both humans and canines. Blue nodes indicate proteins that are members of the top 10 upregulated category.

Protein association network analysis of human data using the STRING-database revealed the greatest number of suggested associations for the proteins, RRM2, KIAA0101, UBE2C, CENPU/MLF1IP, and DTL compared to other members of the top 10 upregulated category. Furthermore, RRM2, KIAA0101, UBE2C, CENPU, and DTL were also found to be associated with each other in both humans and canines (Figure 2D). The human and canine protein association maps of the top 10 upregulated genes are presented in Figure 3. Comparison of the individual human and canine maps demonstrated association similarity ranging from 20 to 80% with an average similarity of 49%. CENPU (80%), KIAA0101 (70%), DTL (60%), SIM2 (50%, 4 canine reported), UBE2C (50%), and LMX1A (50%) demonstrated ≥50% similarity in the suggested protein associations between humans and canine, whereas LBX1 (38%, 8 reported canine), RRM2 (30%), HOXC10 (20%), and IRX5 (20%) demonstrated <50% similarity. Additional STRING analysis of the individual associated proteins reported to be unique to canine or human, suggests that the absence of similarity among the associations of the top 10 upregulated genes may be due to lack of available canine data rather than a true absence of similarity.

KEGG pathway analysis for the individual top 10 upregulated genes was executed using the STRING database. KEGG pathway data was only available for RRM2, UBE2C, DTL, and KIAA0101 in both humans and canines, and only those pathways with a false discovery rate of <0.5 were considered. Common pathways identified as influenced by the associated proteins of these four genes include those involved in DNA replication, nucleotide excision repair, mismatch repair, and base excision repair. Other pathways enriched by proteins associated with KIAA0101, RRM2, and UBE2C include those involved in purine and pyrimidine metabolism and ubiquitin mediated proteolysis, respectively. Despite the presence of unique associated proteins, over 80% of the pathways reported in canine data were also reported in the human data. The KEGG pathways reported for both UBE2C and DTL shared 100% similarity, whereas KIAA0101 and RRM2 demonstrated 88 and 80% similarity between human and canine data, respectively. Proteins associated with RRM2 were reported to be involved in p53 signaling in canines, but this was not reported in the human data. Additionally, RRM2 associated proteins in the human data demonstrated involvement in DNA repair and the cell cycle. Comparison of the percent similarity of the suggested associated proteins and the percent KEGG pathway similarity did not necessarily demonstrate a relationship between the two parameters (Table 1). In fact, RRM2 demonstrated only 30% suggested protein association similarity, while 80% of reported canine pathways were also reported in human data. The other proteins with available pathway data (UBE2C, KIAA0101, DTL), demonstrated >50% similarity in the suggested protein associations as well as >88% similarity in the reported KEGG pathways. The full list of KEGG pathways enriched by the suggested protein associations of the top 10 upregulated genes is presented in the Supplemental Data.

**Table 1:** Comparison of % suggested protein association similarity and % reported KEGG pathway similarity for top 10 upregulated genes.

**Gene**	**Suggested associated protein similarity (%)**	**Reported KEGG pathway similarity**
KIAA0101	70	88%
DTL	60	100%
UBE2C	50	100%
RRM2	30	80%
CENPU	80	No data
LMX1A	50	No data
SIM2	50*	No data
LBX1	38*	No data
IRX5	20	No data
HOXC10	20	No data

* Fewer than 10 suggested associated protein reported in canine data.

*SIM2 (4 canine reported); LBX1 (8 canine reported)*.

### Expression Patterns of the Top 10 Downregulated Genes in Canine ODG and Human Glioma Revealed More Heterogeneity in Human ODG and GBM Than Was Demonstrated by the Top 10 Upregulated Genes

**Figure 4:**
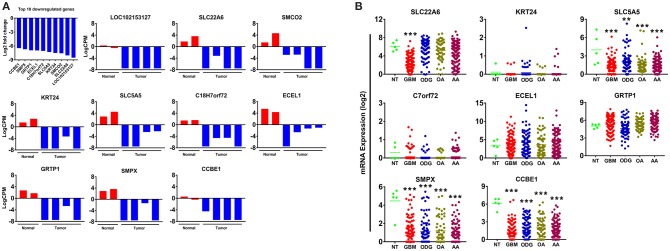
Top 10 downregulated genes in canine oligodendroglioma. **(A)** Expression of the top 10 downregulated genes in individual cases of canine ODG compared to normal brain based on RNAseq data. **(B)** Expression of the top 8 downregulated genes in human gliomas (GBM, ODG, OA, and AA) based on data from Xena Browser (TCGA: low grade glioma and glioblastoma). Two of the top 10 downregulated genes, SMCO2 and LOC102153127, were not available in human data and were, therefore excluded from this dataset (*p ≤ 0.05, **p ≤ 0.01, ***p ≤ 0.001).

Our results demonstrated a −6.5 to −8.5 log2-fold decrease in the mean expression (*p* ≤ 0.002–3.45E-10) of the top 10 downregulated genes (Figure 4A) in canine ODG cases compared to normal brain. These genes demonstrated downregulation consistently across all cases of canine glioma. Evaluation of these genes in human disease demonstrated that 50% (4 out of 8 available) of these genes were also downregulated in human ODG and 63% (5 out of 8 available) in human GBM. The genes, LOC102153127 and C18H7orf72, were found only in canine ODG and were not available in the human database. Figure 4B shows the expression level of the downregulated genes in human ODG, OA, AA, and GBM compared to normal brain tissue (NT). SMPX, CCBE1, and SLC5A5 demonstrated significant downregulation across all subtypes of human glioma with a *p* ≤ 0.0001, whereas SLC22A6 (*p* < 0.0001) and KRT24 (*p* ≤ 0.008) were shown to be downregulated only in human GBM. C7orf72 (Ortholog of Canine C18H7orf72/SPATA48) was not found to be downregulated in human GBM but did demonstrate significant downregulation in human ODG (*p* ≤ 0.01) and OA (*p* < 0.006). ECEL1 and GRTP1 did not demonstrate significant downregulation in any of the subtypes of human glioma. It is important to acknowledge that several of the genes, including CCBE1, LOC102153127 and C18H7orf72, display negligible levels of expression in normal canine brain suggesting that downregulation in canine glioma may not be biologically significant. For this reason, these genes were not confirmed by RT-PCR. However, due to the small sample size as well as the heterogeneity of glioma tumors, we included this data for completeness and further investigation will be needed to determine their significance in both canine and human glioma.

**Figure 5:**
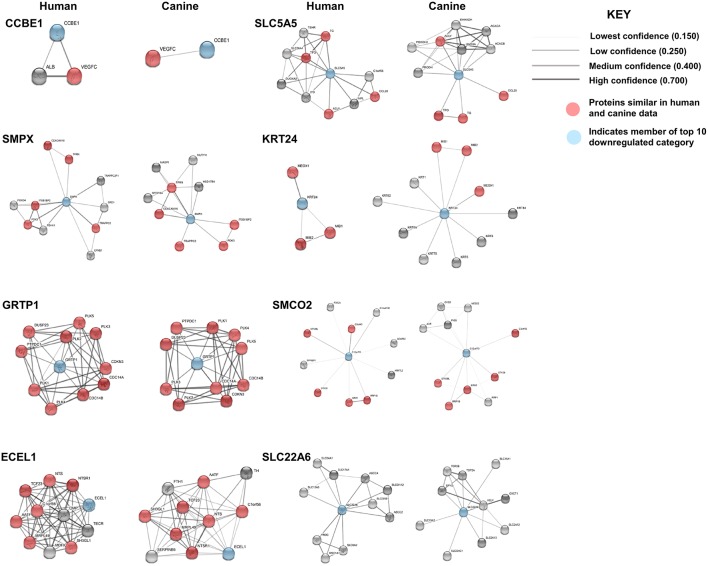
Comparison of the suggested protein associations of the top 10 downregulated genes. Data was collected using the STRING database (functional protein association network version-10-5) at medium confidence (0.400). Increasing confidence is demonstrated by increased thickness and darkness of edges (intersecting lines). Red notes indicate proteins involved in similar associations in both humans and canines. Blue nodes indicate proteins that are members of the top 10 downregulated category.

STRING functional protein association network analysis of the downregulated genes is shown in Figure 5. Comparison of the human and canine gene maps for each of the downregulated genes at medium confidence (0.400), demonstrated association similarity ranging from 0 to 100%, with an average of 48% association similarity. GRTP1 (100%), CCBE1 (100%, 1 canine reported), ECEL1 (70%), SMPX (56%, 9 canine reported), and SMCO2/C12orf70 (50% at low confidence) exhibited ≥ 50% association similarity, whereas SLC5A5 (40%), KRT24 (30%), and SLC22A6 (0%) exhibited <50% similarity. Interestingly, the STRING database reported more associations for KRT24 (11 associations at medium confidence) in the canine data than in the human data. This finding demonstrates how canine data could provide insight into possible associations that could be evaluated in humans in order to further our understanding of glioma pathogenesis. Additionally, evaluation of possible associations between the top 10 up- and downregulated protein gene products, did not yield any evidence supporting associations between members of the two groups.

KEGG pathway analysis for the top 10 downregulated genes was limited by data availability. KEGG pathway data for both humans and canines was available only for the genes, GRTP1 and SLC5A5. Only those pathways with a false discovery rate of <0.5 were considered. Common pathways identified as influenced by the suggested associated proteins of GRTP1 include those involved in the cell cycle and FOXO signaling pathway, whereas the suggested associated proteins of SLC5A5 were found to influence pathways involved in thyroid hormone synthesis and autoimmune thyroid disease. KEGG pathway data was more abundant for the proteins associated with SLC5A5 in canines (18 pathways reported) than in humans (2 pathways reported). The pathways shown to be enriched by the associated proteins from the canine data include those involved in pyruvate, amino acid and fatty acid metabolism. With the exception of GRTP1, the percent similarity of suggested associated proteins did not appear to correspond to the percent similarity of the reported KEGG pathways. This was, perhaps, in large part, due to the lack of available KEGG pathway data for the top 10 downregulated genes in both humans and canines. A comparison of the percent similarity of suggested associated proteins and percent similarity of the KEGG pathways is presented in Table 2. The full list of KEGG pathways enriched by the suggested protein associations of the top 10 downregulated genes is presented in the Supplemental Data.

**Table 2:** Comparison of percent suggested protein association similarity and percent reported KEGG pathway similarity for top 10 downregulated genes.

**Gene**	**Suggested associated protein similarity**	**Reported KEGG pathway similarity**
GRTP1	100%	100%
SLC5A5	40%	11%
CCBE1	100%*	No data
ECEL1	70%	No data
SMPX	56%	No data
SMCO1/C12orf70	50%**	No data
SLC22A6	0%	No data
KRT24	30%	No data
C18H7orf72	No data	No data
LOC102153127	No data	No data

* Fewer than 10 suggested associated proteins reported in canine data; CCBE1 (1 canine reported).

** *Similarity calculated using low confidence data*.

### In Terms of Cluster Differentiation (CD) Gene Upregulation Canine ODG More Closely Resembles Human GBM and Not Human ODG

**Figure 6:**
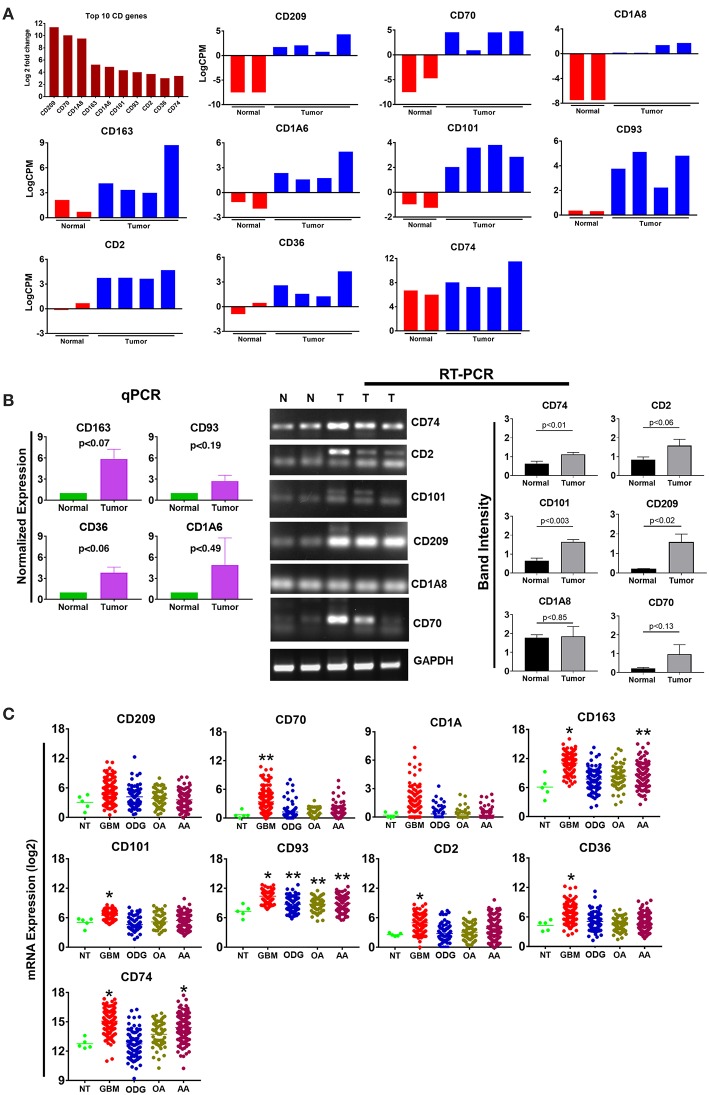
Top 10 cluster differentiation genes in canine oligodendroglioma. **(A)** Expression of the top 10 CD genes canine gliomas based on RNAseq data. **(B)** qPCR/RTPCR analysis confirming the upregulation of the top 10 CD genes in canine ODG. **(C)** Expression of the top 10 CD genes in human glioma subtypes (GBM, ODG, OA, and AA), based on data from Xena Browser (TCGA: low grade glioma and glioblastoma). The isoforms CD1A6 and CD1A8 were reported in canine data, however, data was only available for CD1A in human data (*p ≤ 0.05, **p ≤ 0.01, ***p ≤ 0.001).

Cluster differentiation (CD) molecules serve as cell surface markers used for the characterization of immune cells and are an important component of the anti-tumor immune response. Evaluation of our data demonstrated a 4–12 log2-fold increase in the mean expression (*p*-value ranging from *p* ≤ 0.02 to 4.45E-08) of the top 10 upregulated CD genes in canine ODG cases (Figure 6A). These CD genes were found to be upregulated across all cases of canine gliomas compared to normal brain. We confirmed the overexpression of these genes using qPCR/RT-PCR (Figure 6B). qPCR was used to confirm the expression of CD163 (*p* = 0.07), CD36 (*p* = 0.06), CD93 (*p* = 0.19), and CD1A6 (*p* = 0.49). RT-PCR data was conducted using the average expression of the canine tumors combined and demonstrated significant upregulation of CD101 (*p* = 0.0032), CD209 (*p* = 0.0206), and CD74 (*p* = 0.0166), but not CD2 (*p* = 0.0616), CD1A8 (*p* = 0.8518), or CD70 (*p* = 0.1361). As previously discussed in relation to the top 10 upregulated genes, this lack of significant upregulation demonstrated by the RT-PCR data is likely attributed to the small tumor sample size and tumor heterogeneity, which is demonstrated by the variable expression of these genes as shown in Figure 6A. It should also be noted that the expected PCR fragment size of CD209 (231 bp) and CD1A8 (162 bp) were different from the observed fragment size, raising the question of whether the observed fragment represents a canine specific isoform or transcript variant (Figure 6B). Additional studies will be needed for target validation and evaluation of this observed difference. Our investigation of these top 10 differentially expressed cluster differentiation (CD) genes in human glioma revealed that 11% (1 out of 9 available) were upregulated in human ODG and 80% (7 out of 9) were upregulated in human GBM (Figure 6C). It should be noted that the *CD1A* isoforms, *CD1A6* and *CD1A8*, were reported individually in canine ODG, however, human data was only available for *CD1A. CD93* demonstrated significant upregulation in all human glioma subtypes: GBM (*p*-value 0.0001), ODG (*p*-value 0.03), OA (*p*-value 0.02), and AA (*p*-value 0.01). CD163 and CD74 were found to be significantly upregulated in human GBM and AA, but not human ODG or OA (*p*-value 0.001), whereas *CD70, CD2, CD101*, and CD36 were significantly upregulated only in human GBM (*p*-value 0.001). *CD209* and *CD1A* did not demonstrate significant upregulation in any of the human glioma subtypes.

**Figure 7:**
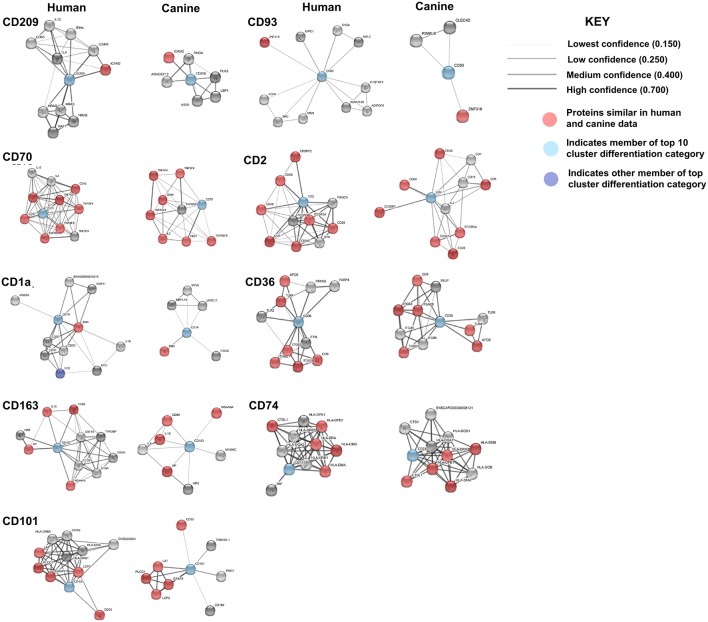
Comparison of the suggested protein associations of the top 10 downregulated genes. Data was collected using the STRING database (functional protein association network version-10-5) at medium confidence (0.400). Increasing confidence is demonstrated by increased thickness and darkness of edges (intersecting lines). Red notes indicate proteins involved in similar associations in both humans and canines. Light Blue nodes indicate proteins that are members of the top 10 CD category. Dark blue nodes indicate proteins that are members (other than the CD gene being evaluated) of the CD gene category.

Comparison of the canine and human association maps demonstrated association similarity ranging from 17 to 88%, with an average similarity of 50% (Figure 7). CD70 (88%, 8 canine reported), CD2 (70%), CD101 (63%, 8 canine reported), CD36 (60%), CD163 (57%, 7 canine reported), and CD74 (50%) demonstrated ≥50% association similarity, whereas CD93 (33%, 3 canine reported), CD1A (20%, 5 canine reported), and CD209 (17%, 6 canine reported) demonstrated <50% association similarity. It should be noted that the data available on the canine CD genes was limited in the STRING database and, as stated previously, the lack of available data may play a large role in the absence of association similarities among the genes evaluated in this study. As can be seen from the above data, CD genes exhibiting the lowest level of similarity to human data are also those CD molecules with the fewest reported suggested protein associations (CD1A, CD209, and CD93) in canine data.

KEGG pathway analysis using the STRING database revealed pathway data for both canines and humans for all CD genes except, CD93 and CD1A. CD1A KEGG pathway analysis was available in human data, while CD93 data was not available in either species. This is not surprising given the lack of available canine protein association data of these two CD genes. Human KEGG pathway data of CD1A suggested that associated proteins influenced pathways involved in T-cell receptor signaling and response to pathogens such as trypanosomiasis, measles, and HTLV-1. Generally speaking, those CD genes reported to have a suggested associated protein similarity of >50% (CD70, CD2, CD101, CD36, CD163, and CD74), also demonstrated the greatest reported KEGG pathway similarity (Table 3). KEGG pathway analysis of the proteins associated with CD70, CD101, CD74, and CD209 revealed that 100% of the reported pathways in canine data were also reported in human data. Interestingly, CD74 demonstrated only 50% suggested associated protein similarity, yet all 20 KEGG pathways reported in canine data were also reported in human data. Overall, the KEGG pathways found to be enriched by proteins associated with the protein products of the top 10 CD genes in both humans and canines include those involved in autoimmunity, response to a variety of pathogens, T cell interactions, inflammation, and cancer. The full list of KEGG pathways enriched by the suggested protein associations of the top 10 CD genes is presented in the Supplemental Data.

**Table 3:** Comparison of percent suggested protein association similarity and percent reported KEGG pathway similarity for top 10 cluster differentiation genes.

**Gene**	**Suggested associated protein similarity (%)**	**Reported KEGG pathway similarity**
CD70	88*	100%
CD2	70	55%
CD101	63*	100%
CD36	60	57%
CD163	57*	40%
CD74	50	100%
CD209	17*	100%**
CD93	33*	No data
CD1A (CD1A6 and CD1A8 in canine)	20*	No data

* Fewer than 10 suggested associated proteins reported in canine data; CD70 (8 reported); CD101 (8 reported); CD163 (7 reported); CD93 (3 reported); CD1A (5 reported); CD209 (6 reported).

** *Only one KEGG pathway was reported in canine data*.

### Cell Cycle Regulation Genes Such as KIF11 and UBEC2C Are Highly Dysregulated in Both Canine and Human MG and Can Serve as Potential Therapeutic Targets

**Figure 8:**
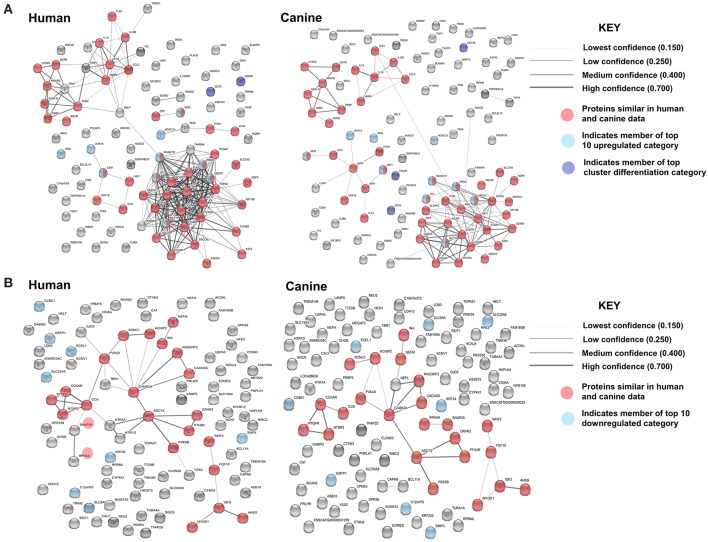
Comparison of the suggested protein associations for the top 100 up- and downregulated genes in human and canine data. **(A)** Comparison of suggested protein associations of the protein products of the top 100 upregulated genes between humans and canines. There were 99 proteins available in the canine data and 93 proteins available in human data. Forty seven proteins were involved in similar associations between human and canine data (red nodes). Light blue nodes indicate members of the top 10 upregulated gene category. Dark blue nodes indicate members of the top 10 CD gene category. **(B)** Comparison of suggested protein associations of the protein products of the top 100 downregulated genes between humans and canines. There were 96 proteins available in the canine data and 90 proteins available in human data. Twenty one proteins were involved in similar associations between human and canine data (red nodes). Light blue nodes indicate members of the top 10 downregulated gene category.

In order to evaluate the major pathways enriched by the differentially expressed genes in canine glioma, we expanded our gene set and performed protein association analysis for the top 100 genes found to be up- and downregulated in canine ODG (Figure 8). As is evident by the edge density in the top 100 upregulated human protein association map (Figure 8A top left) compared to the canine map (Figure 8A top right) more suggested protein associations have been characterized in the human data than in canine. Despite the lack of available data, our STRING database inquiry demonstrated that 47% of the top 100 upregulated proteins and 24% of the top 100 downregulated proteins shared similar associations in human and canine data (Figures 8A,B).

Evaluation of both KEGG and Biological Processes GO pathways was executed for the top 100 up- and downregulated genes. A single KEGG pathway was reported to be enriched by genes from the top 100-downregulated categories in both canines and humans. Eight KEGG pathways were reported to be enriched by members of the top 100-upregulated genes in canine data, six of which were also reported in human data, indicating 75% reported KEGG pathway similarity. The KEGG pathways reported in both canine and human data include those involved in type 1 diabetes mellitus, cytokine-cytokine receptor interaction, graft-vs.-host disease, inflammatory bowel disease, toll-like receptor signaling, and rheumatoid arthritis. In human data, the p53 pathway was reported to be enriched by members of the top 100-upregulated genes, but this finding was not reported in canine data. A full list of the reported KEGG pathways and the specific genes involved are reported in the Supplemental Data.

Pathway analysis was then performed to determine the significant biological process GO pathways influenced by members of the top 100 up- and downregulated gene category in both humans and canines. Biological process GO data for the top 100-downregulated genes was not available in the STRING database for canines. Human data, however, revealed that the 12 reported pathways influenced by members of the top 100-downregulated gene category included those involved in cell-to-cell signaling, ion and transmembrane transport, synaptic transmission, and neurofilament assembly. Details of these pathways and the genes involved are presented in the Supplemental Data.

**Figure 9:**
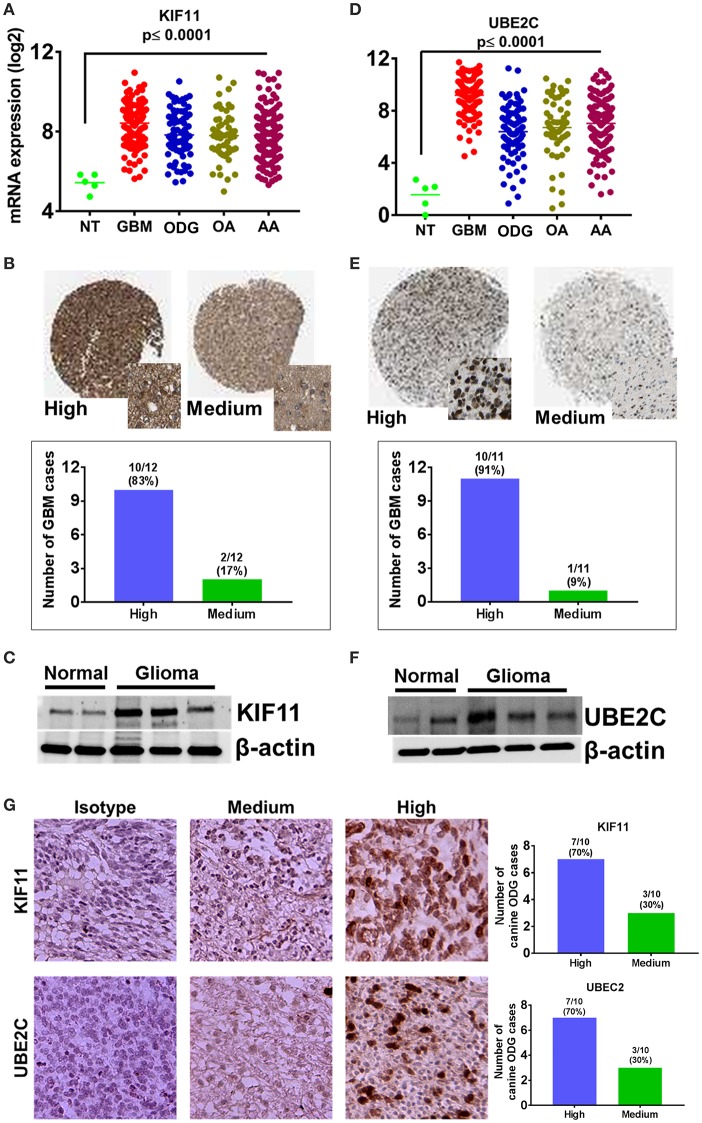
Overexpression of cell cycle regulators KIF-11 and UBE2C in human and canine glioma. **(A)** Overexpression of KIF-11 mRNA (TCGA data) in human GBM, ODG, OA, and AA tumors. **(B)** KIF-11 protein levels were upregulated in human GBM (Human Protein Atlas). **(C)** Western blot analysis showed the overexpression of KIF-11 in canine ODG compared to normal brain. **(D)** UBE2C mRNA overexpression (TCGA data) in human GBM, ODG, OA, and AA tumors. **(E)** UBE2C protein levels were overexpressed in human GBM (Human Protein Atlas). **(F)** Western blot analysis showed the overexpression of UBE2C in canine ODG as compared to normal brain. **(G)** IHC analysis of KIF-11 and UBE2C in canine ODG cases (*n* = 10). Specific staining intensity was classified as high with strong signal with 100% cells positivity and medium as moderate to strong signal with 60–80 percent cells positivity. Quantification of high and medium expression cases were performed and presented as percent cases.

A total of 128 biological pathways were reported to be influenced by members of the top 100-upregulated gene category for humans, whereas only 11 pathways were available in canine data. Pathways identified as being influenced by members of the top 100-upregulated gene category in humans included those involved in the cell cycle, cellular metabolism, neurogenesis, and immune response. A complete list of the biological process GO pathways and the specific genes involved are presented in the Supplemental Data. We evaluated the 11 pathways reported for canines in the human data and found available data for 6 of the 11 pathways. From this list of 6 matched pathways, we included only the pathways with a false discovery rate of <0.02 in human data and <0.05 in canines. This resulted in selection of three pathways reported in both human and canines, which are presented in Table 4. Several cell cycle regulatory genes are significantly upregulated in canine OG (Figure 2), however, we selected KIF11 and UBE2C, which are commonly upregulated in canine and human glioma, for further evaluation at the protein level. Our results presented in Figure 9 demonstrate the overexpression of both KIF11 and UBE2C mRNA (TCGA data) and protein (The Human Protein Atlas) in human gliomas as well as in canine ODG (Figures 9A–G).

**Table 4:** Biological process pathway GO influenced by top 100 upregulated genes in both canines and humans.

**Biological process pathway GO ID**	**Pathways description**	**Top 100 upregulated genes involved (human)**	**FDR (human)**	**Top 100 upregulated genes involved (canine)**	**FDR (human)**
GO.0045086	Positive regulation of Interleukin-2 biosynthesis process	CD28, IL1A, IL1B	0.00655	IL1A, IL1B	0.0374
GO.0007346	Regulation of mitotic cell cycle	CCNB1, CCNB3, CD28, DLGAP5, IL1A, IL1B, **KIF11**, Top2A, Tp63, **UBE2C**	0.00679	CCL3, IL1A, IL1B	0.0374
GO.0010564	Regulation of cell cycle process	CCNB1, CCNB3, CD28, DLGAP5, IL1A, IL1B, **KIF11**, TPLK4, Tp63, **UBE2C**	0.0128	CCNB3, IL1A, IL1B	0.0495

## Discussion

The incidence of intracranial neoplasms in canines is estimated to be between 2 and 4.5% based on necropsy-dependent survey (15). The most common types of primary brain tumors reported in dogs include meningiomas (30–45%) and gliomas (astrocytomas (excluding glioblastoma)−17%, oligodendrogliomas −14%, and glioblastoma −3%), with specific breeds, such as the Boston Terrier, Golden Retriever, Boxer, French Bulldog, Miniature Schnauzer, and Rat Terrier, demonstrating a significantly increased risk of developing primary intracranial neoplasms (15, 16). A detailed discussion of the various types of canine brain tumors is beyond the scope of this paper, however, such a review can be found elsewhere (16).

The interest in the utility of the canine model of glioma has become so great that the National Cancer Institute's Comparative Oncology Program created the Comparative Brain Tumor Consortium (CBTC), whose mission is to integrate the use of canine brain tumors into all facets of brain tumor research in order to develop novel therapies and diagnostic strategies (15, 17). Most recently, the CBTC has published a defined set of uniform criteria to be used for the evaluation, diagnosis, and grading of canine gliomas (astrocytomas, oligodendrogliomas) that will allow for better comparison to human glioma (17).

This strong interest in the canine model of glioma can be attributed to the many studies that have previously demonstrated the similarities between human and canine primary brain tumors (4, 5, 18, 19). These studies have demonstrated that canine brain tumors exhibit histopathological, immunological, molecular, and clinical characteristics similar to those of human disease, however there is no breed specific difference in the gene expression profile (4, 20, 21). More specifically, recent studies have shown that the expression pattern of key signaling pathway proteins, MDM2, TP53, PTEN, P21, AKT, RB1, mTOR, and MAPK is similar in both human and canine gliomas (4, 5). Additionally, our group recently demonstrated that, like human MGs, canine ODGs also exhibit dysregulation of PI3K, RAS, PIK3CB, TP53, SOX2, DNMT1, CDK4, CCNB1, ERBB2, OLIG2, and PAK1 (4). Despite the numerous similarities, studies evaluating the presence of IDH mutations and 1p/19q co-deletions in canine brain tumors suggest that these are not major events that occur in canines, but are important characteristics of human disease (18, 19, 22).

In this study, we demonstrate that the top 10 genes found to be upregulated in canine ODG contribute to cancer treatment resistance [*RRM2* (23), *KIAA0101* (24), *HOXC10* (25, 26)], migration [*KIAA0101* (24, 27)], invasion/metastasis [*LBX1* (28), *HOXC10* (29–31), *SIM2* (32–34)], and cancer cell survival [*RRM2* (35, 36), *IRX5* (37, 38), *UBE2C* (39)]. Several of these genes are also reported to serve as prognostic markers [*RRM2* (40), *UBE2C* (41), *DTL* (42)] or to be associated with recurrence [*LMX1A* (43)] in other types of cancer. We observed similar expression patterns of these genes in humans and canines, with 80% of the genes upregulated in canine ODG also upregulated in human ODG and 90% of them are upregulated in human GBM. The observed gene expression pattern of the upregulated genes seen in our study suggests that canine ODG more closely resembles human GBM rather than human ODG or other glioma subtypes. Additionally, we report an average of 49% suggested associated protein similarity, as well as >80% similarity in the reported KEGG pathways. Our STRING database inquiry revealed that the protein products of *UBE2C, CENPU*/*MLF1IP, KIAA0101, RRM2*, and *DTL* were found to be associated with one another in both humans and canines. This pattern was not demonstrated by members of the top 10 downregulated or CD gene categories, providing insight into the importance of the network of the upregulated genes in glioma pathogenesis.

The genes *RRM2, UBE2C, LMX1A, HOXC10*, and *SIM2* have previously been evaluated in glioma and are shown to protect glioma cells from replication stress, DNA damage, and apoptosis [*RRM2* (36)], to be associated with decreased overall length of survival and increased tumor aggressiveness [*UBE2C* (41)], to contribute to tumor immunosuppression [*HOXC10* (31)] and to promote glioma migration and invasion [*HOXC10* (31), *SIM2* (32, 34)]. Previous attempts to target these genes have resulted in decreased viability and tumor initiating capacity of glioma cells [*RRM2* (36)], induction of autophagic glioma cell death [*UBE2C* (39)], promotion of apoptosis, inhibition of cellular proliferation [*HOXC10* (31)], and decreased tumor invasion [*HOXC10* (31), *SIM2* (34)]. The remaining members of the top 10-upregulated genes, *IRX5, KIAA0101, LBX1*, and *DTL*, have been studied in other cancers, but have not been well-studied in glioma.

In prostate cancer, IRX5 knockdown is associated with increased p53 and p21 expression, G2-M arrest, and increased apoptosis (44). In tongue squamous cell carcinoma, overexpression of IRX5 was found to contribute to proliferation, migration, and invasion of cancer cells via activation of the NF-kB pathway and interactions with osteopontin (45). Interestingly, 1,25-dihydroxyvitamin D3 has been shown to negatively regulate IRX5 expression in both androgen-sensitive prostate and estrogen-sensitive breast cancer cell lines (44). Myrthue et al. also demonstrated decreased expression of IRX5 in human prostate cancer samples from patients who received weekly high-dose 1,25-dihydroxyvitamin D3 prior to radical prostatectomy (44). This finding may have interesting implications for targeting IRX5 overexpression in glioma patients as 1,25-dihydroxyvitamin D3 is known to traverse the blood brain barrier (46). Increased KIAA0101 expression has been demonstrated in the peripheral blood mononuclear cells of patients with hepatic cancer, suggesting a role for KIAA0101 as a predictive biomarker (47). KIAA0101 has also been shown to be associated with tumor growth, migration, and invasion in other types of cancer (24, 27, 48–50). Its inhibition results in the suppression of cell growth and G1/S transition via the upregulation of p53 and downregulation of CCNE2, CDK6, and CDKN1A (24, 27, 48–50). In breast cancer, Yu et al. reported that LBX1 directs expression of ZEB1, ZEB2, Snail1, and TGF-ß2, which are known inducers of the epithelial-to-mesenchyme transition (28). Lastly, overexpression of DTL has been shown to contribute to lymphatic invasion, tumor depth, recurrence, and poor outcomes in gastric carcinoma (42).

With respect to the genes found to be the most downregulated in canine ODG, the similarity between human and canine disease is less pronounced, with only 50% of the genes downregulated in human ODG and 63% downregulated in human GBM. Additionally, we demonstrated an average of 48% similarity in the suggested protein associations of the top 10-downregulated genes between the two species. KEGG pathway analysis of the top 10-downregulated genes was limited by the lack of data in both species, with data reported for only two genes, GRTP1 and SLC5A5, which demonstrated 100 and 11% similarity, respectively. Genes in the top 10 downregulated category are found to act as tumor suppressors [SMCO2 (51), CCBE1 (50)], increase responsiveness to chemotherapy [SLC22A6 (52–54), SLC5A5 (55)], serve as prognostic markers [CCBE1 (56)], and play a role in cellular differentiation [KRT24 (57), CCBE1 (50)]. In other cancers, decreased expression of these genes has been associated with vascular invasion and increased tumor aggressiveness [SLC5A5/NIS (58)], increased proliferation [SMOC2 (51)], increased migration [SMOC2 (51), CCBE1 (59, 60)], and poor prognosis [ECEL (61)]. To the best of our knowledge, KRT24, GRTP1, SLC22A6/OAT1, and SMPX have not been well-studied in glioma. While KRT24, itself, has not been studied in the context of glioma, it has been experimentally determined to be associated with MIB1 and MIB2, which are involved in Notch signaling pathway in brain tumors (62–64). MIB1 also plays a role in angiogenesis (62) and MIB2 may be a positive regulator of NF-kB signaling (65), both of which are important in glioma pathogenesis. Similarly, our STRING database inquiry suggests an association of GRTP1 with members of the polo kinase family, Plk1-5. In previous studies, Plk1 inhibition has been shown to block mitosis and promote apoptosis in cells with high mitotic index and its overexpression has been linked to resistance to chemotherapy (66, 67). Plk1 inhibitors, such as volasertib, have demonstrated promising results in clinical trials (66). In glioma, Plk1 inhibition has been shown to arrest cells in the mitotic phase of the cell cycle (68, 69), impair proliferation, migration, and invasion and to induce apoptosis (70). Evaluation of the GRTP1-Plk interaction utilizing the STRING database molecular action function indicated a regulatory role for GRTP1, suggesting the need for further investigation of GRTP1 as a potential target for anti-glioma therapy.

In addition to the role of genetic alterations in glioma pathogenesis, the contribution of the immune system has been previously well-described (71, 72). In our previous study, we demonstrated similar immunologic profiles of the human and canine glioma microenvironments (4). Expanding upon this, we evaluated the expression of cluster differentiation genes in both humans and canines. We demonstrated that the top 10 CD genes upregulated in canine glioma are involved in influencing immune response through stimulation of dendritic cell-T cell interactions [CD209 (73–75) CD101 (76, 77)], T-cell activation [CD70 (78), CD101 (76, 77), CD2 (79–81)], the promotion of an immunosuppressive tumor microenvironment [CD70 (82), CD163 (83, 84)], stimulation of angiogenesis [CD163 (83, 84), CD93 (85, 86) CD74 (87, 88)], and regulation of monocytes and macrophages [CD36 (89, 90)]. Additionally, several of these CD genes have been previously been selected as targets for experimental treatments [CD70 (78),CD163 (83, 91), CD2 (92), CD36 (93)]. Evaluation of the CD genes differentially expressed in canine ODG demonstrated that 11% of these genes were also upregulated in human ODG and 80% were upregulated in human GBM, suggesting the immune response in canine ODG more closely resembles that of human GBM rather than human ODG. In glioma, these genes are involved in mediating tumor migration [CD70 (82)], angiogenesis [CD93 (94), CD36 (93, 95)], T-cell activation [CD2 (92)], maintenance of glioma stem cell populations [CD36 (96)], tumor proliferation [CD74 (97)], and TMZ resistance [CD74 (97)]. Additionally, CD70 is responsible for promoting macrophage infiltration (82), while CD163 has been shown to drive their M2 polarization (98). The co-expression of these two molecules is associated with decreased survival in GBM patients (82).

Several of these CD genes have previously been evaluated as potential therapeutic targets in GBM. Ge et al. targeted CD70 using chimeric antigen receptor T-cells and their most recent study demonstrated regression of established xenograft in syngeneic GBM mice models through the use of CD70-CAR-T therapy (82). Studies have also shown that the administration of T11TS, a known ligand of CD2, resulted in positive modulation of CD2-associated proteins and stimulation of T-cell activation (92). Recently, Chaudhuri et al. demonstrated that T11TS administration relieved the glioma-induced suppression of the PI3K-AKT pathway in T-cells, which may result in decreased T-cell apoptosis and increased T-cell survival in the glioma microenvironment (99). Similarly, binding of CD36 to its ligands, vasculostatin and trombospondin-1, results in the inhibition of angiogenesis and induction of endothelial cell apoptosis (93, 95). Ghoochani et al. demonstrated that inhibition of MIF-CD74 signaling leads to increased IFNγ release, resulting in glioma growth inhibition and induction of M2 to M1 polarization of glioma-associated microglia (100). In addition, Kitange et al. showed that CD74 knockdown with shRNA reduced activation of Akt and ERK1/2, decreased proliferation and increased the sensitivity of glioma cells to TMZ (101).

Finally, pathway enrichment analysis of the top 100 up- and downregulated genes also demonstrated similarities between human and canine glioma. KEGG pathway analysis of the top 100 genes found to be up- and downregulated in canine ODG, demonstrated 63% similarity in the pathways reported to be enriched by members of the top 100 upregulated gene category and 100% similarity in the pathways enriched by members of the top 100 downregulated category. A single pathway, neuroactive ligand-receptor interaction, was reported to be enriched by members of our specific downregulated gene category in both humans and canines. The specific genes found to be involved included, CCKAR, CRHR2, GABRD, HTR1A, HTR1D, NTSR2, PRLHR, PTGFR, PTH2R, and were identical in both species. Intuitively, the downregulation of these genes makes sense as they are involved in the signaling of neuroactive ligands such as serotonin, neurotensin, CCK, cortisol, prolactin, and PTH, which one would expect to be important in normal brain function, but not necessarily in a tumor composed primarily of malignant glial cells. KEGG pathways important to cancer pathogenesis and enriched by members of the top 100-upregulated gene category, in both humans and canines, include those involved in the toll-like receptor signaling pathway and cytokine-cytokine receptor interaction. The specific genes from our study found to enrich these pathways were identical in humans and canines and include CD70, IL-18RAP, IL-1A, PPBP/CXCL7, TLR1, IL-12β, IL-1β, and CCL3. With respect to the toll-like receptor signaling pathway, activation of TLR1 results in increased production of inflammatory cytokines including IL-1β and IL-12β. The contribution of IL-1β to the progression of human glioma by influencing proliferation, migration, and invasion as well as the development of the tumor microenvironment has been previously well-documented (102, 103). IL-12β is not as well-studied in glioma, however, studies evaluating its effect on T-cells in other settings have demonstrated that prolonged exposure to IL-12 induces T-cell exhaustion, a phenomenon recently shown to be quite severe in glioblastoma (104, 105). A discussion of the pathophysiological role of cytokines in glioma is reviewed elsewhere (106), and the role of several of the specific genes demonstrating enrichment of the cytokine-cytokine receptor interaction KEGG pathway has previously been discussed (CD70, IL-12β, IL-1β). The remaining genes (IL-1α, CXCL7, IL-18RAP, CCL3) have not been well-studied in glioma, with the exception of CCL3, which is shown to improve the response to dendritic cell vaccines of patients with GBM (107).

Evaluation of biological process GO pathways enriched by members of the top 100 downregulated gene category demonstrated that these genes influence pathways involved in cell-to-cell signaling, ion and transmembrane transport, synaptic transmission, and neurofilament assembly. This is not surprising given that malignant gliomas are composed primarily of malignant glial cells, which would have little use for genes responsible for the normal structure and function of neurons. Conversely, our analysis of the biological process GO pathways enriched by members of the top 100 upregulated gene category in both humans and canines revealed that these genes collectively influence pathways involved in the cell cycle, cellular metabolism, and immune response. We demonstrated the enrichment of three common pathways reported for both canines and humans: (1) positive regulation of IL-2 biosynthesis (GO.0045086), (2) mitotic cell cycle regulation (GO.0007346), and (3) the regulation of cell cycle processes (GO.0010564). The specific genes involved in these pathways include, CCNB1, CCNB3, CD28, DLGAP5, IL1α, IL1β, KIF11, PLK4, TP63, and UBE2C. In our previous study, we showed that CCNB1 and KIF11 are upregulated in human and canine glioma (4). In the current study, we show that CCNB3, IL-1β, IL-1α, and UBE2C are also upregulated in both species. IL-1β and IL-1α were found to play a role in all three of the matched pathways in both humans and canines (Table 4). CCNB1 and CCNB3, which encode mitosis-related cyclins, were involved in two of the matched pathways (GO.0007346 and GO.0010564) in humans, but CCNB3 was only shown to be involved the regulation of cell cycle process in canines (GO.0010564). KIF11 and UBE2C are involved in the regulation of the mitotic cell cycle (GO.0007346) and the regulation of cell cycle process (GO.0007346) pathways in humans, however, due to a lack of data we were not able to evaluate their contribution to these pathways in canines. In GBM, KIF11 has previously been reported to be involved in cell survival and stem cell growth (108), as well as the promotion of invasion, proliferation, and self-renewal (109). The inhibitors, FAK inhibitor Y15 (110), Monastraol and Ispinesib have been found to target KIF11 in GBM, and KIF11 inhibition has been shown to prolonged the survival of mice bearing orthotropic GBM (109). While the KIF11 inhibitor, Ispinesib, has already been tested in a phase I clinical trial in pediatric patients with recurrent/refractory tumors and demonstrated positive therapeutic response in three patients with disease stabilization in an additional patient, it has not been evaluated in GBM in clinical trials (111, 112). Additionally, UBE2C has been associated with decreased overall length of survival and increased tumor aggressiveness in GBM (13, 41, 113, 114). Inhibition of UBE2C in GBM has shown to result in the induction of autophagic death in glioma cells, but its inhibition has not been evaluated in GBM in clinical trials either (39). Human Protein Atlas data, shows that 83% of glioma patients express moderate to high levels of KIF11 and 90% of patients express at least some level of UBE2C. Targeting KIF11 and UBE2C using small molecule inhibitors Ispinesib and Monastrol (KIF11 inhibitors) and NSC697923 (UBE2C inhibitor) in the canine model of glioma can potentially be a promising therapeutic strategy for future evaluation in human GBM.

In summary, we further characterized the molecular derangements of canine MG and provide an in-depth comparison of the dysregulated genes in canine and human MG. This study provides further support for the use of spontaneous canine glioma as a transitional model for use in bridging the gap between the pre-clinical mouse model and clinical benefit for human glioma patients. While additional experimental work is needed based on the results of this preliminary study, we have demonstrated that there is significant similarity between human and canine glioma with respect to the expression pattern of glioma-promoting genes, suggested associated proteins and pathway enrichment. Several of the previously discussed, common genes may be candidates for therapeutic targeting, thus allowing for the successful translation to clinical benefit for human glioma patients.

## Data Availability

The data analyzed in this study can be found at http://www.ncbi.nlm.nih.gov/bioproject/557484 with SubmissionID: SUB6093652 and BioProject ID: PRJNA557484.

## Author Contributions

DM, SC, KF, MH, and BT: data generation and data analysis. DM, SC, and MD: data analysis and manuscript preparation. SC, AA, RB, and MD: conception, data oversight, and contribution of experimental materials. MD: conception, contribution of experimental materials, manuscript preparation, and overall supervision of the project.

### Conflict of Interest Statement

The authors declare that the research was conducted in the absence of any commercial or financial relationships that could be construed as a potential conflict of interest.
