# Supplementary material for: Common Molecular Alterations in Canine Oligodendroglioma and Human Malignant Gliomas and Potential Novel Therapeutic Targets
Source: Front Oncol. 2019 Aug 14;9:780. doi: 10.3389/fonc.2019.00780 (PMC6702544; doi:10.3389/fonc.2019.00780)
Supplement: Supplementary file 1 [file Data_Sheet_1.PDF]

Common molecular alterations in canine and human malignant gliomas and potential novel therapeutic targets

Dana Mitchell MS[1], Sreenivasulu Chintala PhD[1], Kaleigh Fetcko MD[1], Mario Henriquez BS[1], Brij N. Tewari Ph.D[1], Atique Ahmed PhD[3], R Timothy Bentley DVM[2] and Mahua Dey MD[1]*

[1] Department of Neurosurgery, Indiana University School of Medicine, Simon Cancer Center, Indiana, USA

[2] Department of Veterinary Clinical Sciences, Purdue University Center for Cancer Research, Purdue University, West Lafayette, Indiana, USA

[3] Department of Neurological Surgery, Northwestern University, Chicago, Illinois, USA

Figure S1

A. **RT-PCR of Upregulated Genes Blots**

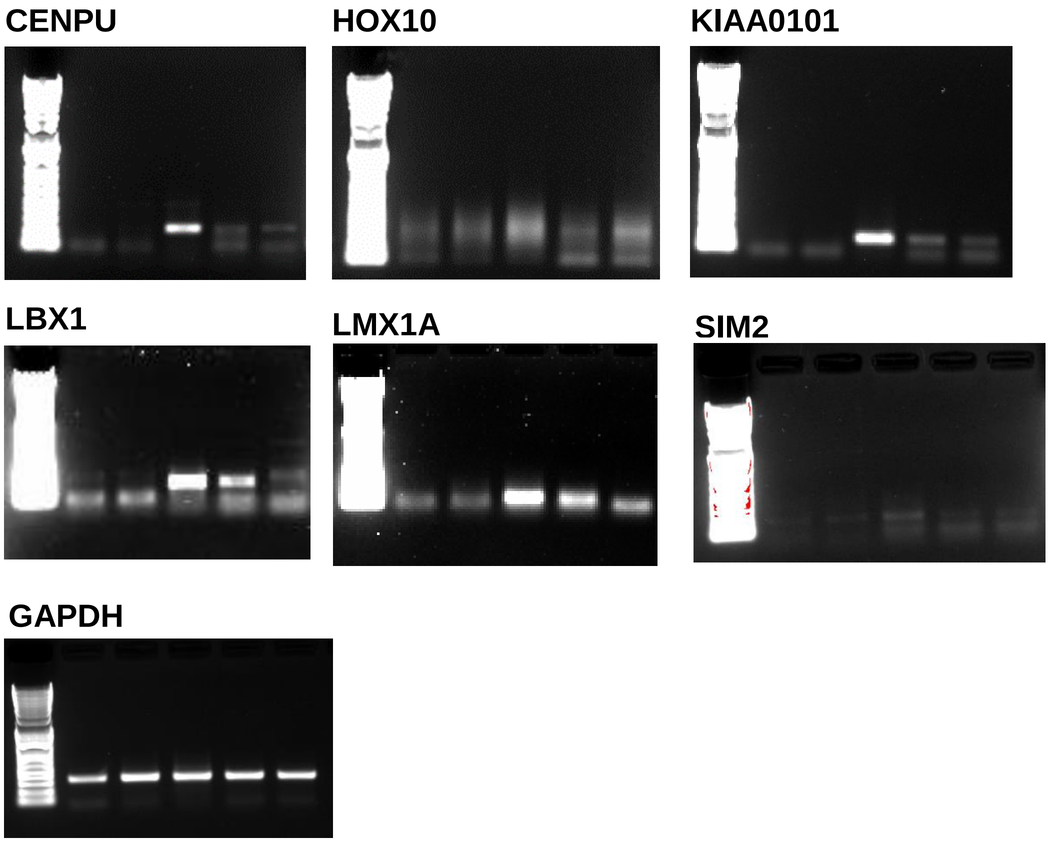

B. RT-PCR of CD Gene Blots

CD74

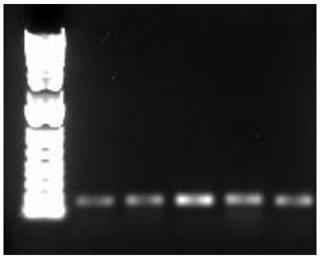

CD2

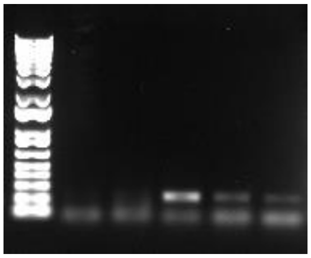

CD101

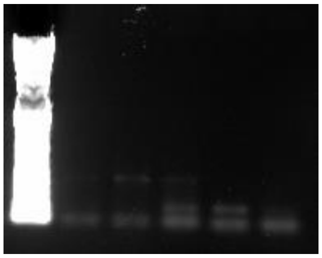

CD209

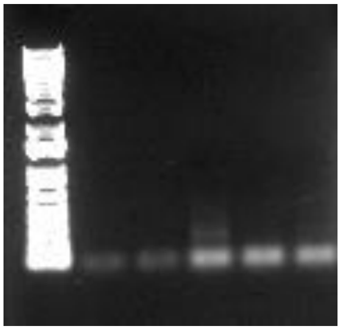

CD1A8

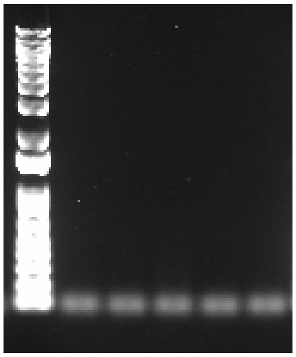

CD70

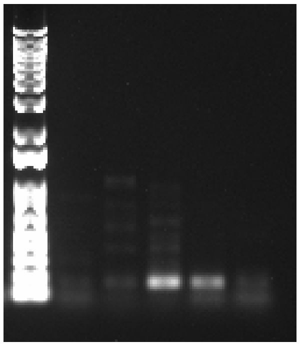

GAPDH

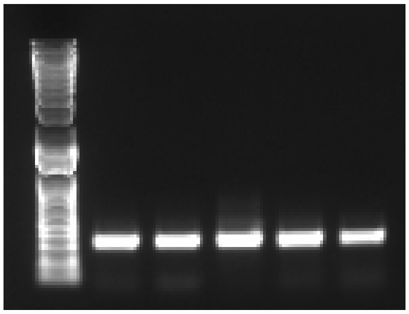

C. Western Blots of KIF11 and UBE2C

KIF11

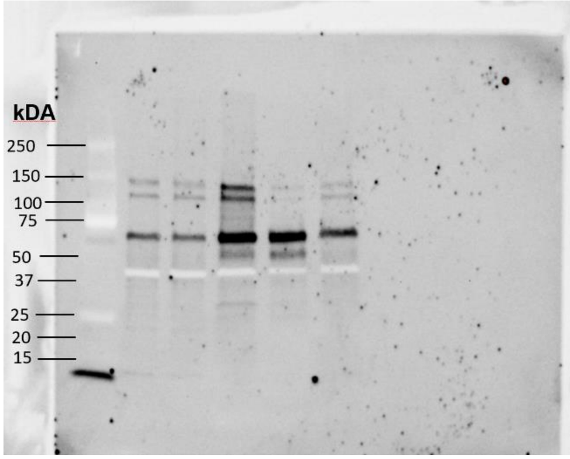

Beta-Arrestin

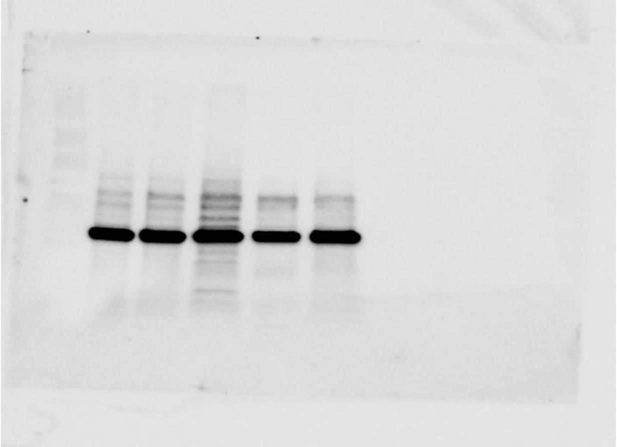

UBE2C

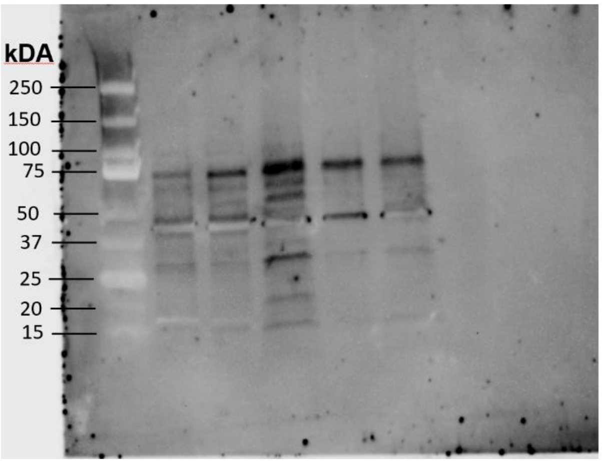

Beta-Arrestin

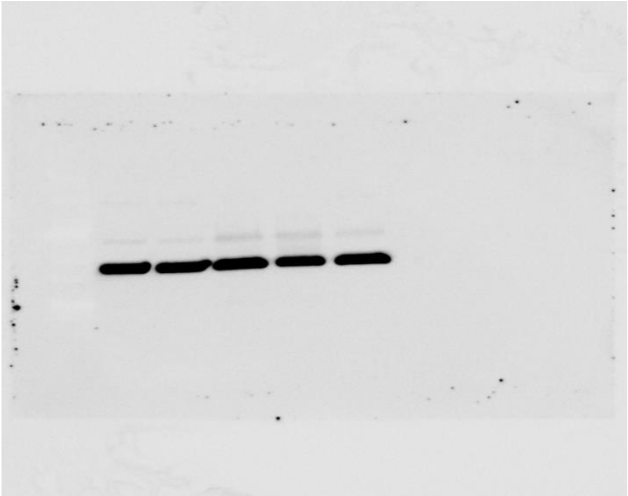

D. Primers

D-GAPDH: F: 5’-ATC ACC ATC TTC CAG GAG CGA GA-3’
D-GAPDH: R: 5’-GTC TTC TGG GTG GCA GTG ATG G-3’

D-RRM2-F: 5’- GTTACCCCTGCTTTCTCCCT- 3’
D-RRM2-R: 5’-AGACAGCCACCGTTACAGAA-3’

D-IRX5-F: 5’-CAAGGGCGAGAAGATCATGC- 3’
D-IRX5-R: 5’-CGATGTTCTCCTCCTCCTCC-3’

D-KIAA0101-F: 5’-GTTAAACACCCTCGGTTGGA-3’
D-KIAA0101-R: 5’-GATGAAAGCGGTGTGGAGTT-3’

D-LBX1-F: 5’-AAGACCTTTAAGGGGCTGGA-3’
D-LBX1-R: 5’-CGATTCTGGAACCAGGTGAT-3’

D-HOXC10-F: 5’-AGTGTGGCTGGTGTGTGTGT-3’
D-HOXC10-R: 5’-GAAATGCAGCAAGGAAGGAG-3’

D-SIM2-F: 5’-TGTGGAAAACAGTGGTGGAA-3’
D-SIM2-R: 5’-TCTTGCTGAACCAACAGTGC-3’

D-LMX1A-F: 5’-TCTAAGCCGTGCAGAAAGGT-3’
D-LMX1A-R: 5’-TGAGTGTTCTGCTGGTCCTG-3’

D-DTL-F: 5’-GATGGCTCACTGGAATGCTG- 3’
D-DTL-R: 5’-TTGCCATCTCTTCCACCTGT-3’

D-CENPU-F: 5’-CAGCATCTGCTTGAACTCCA-3’
D-CENPU-R: 5’-GGGCTCTCGCTGTCACTAAC-3’

D-CD209-F: 5’-ACTCTTCCAGGAAAGCTGCT- 3’
D-CD209-R: 5’-CTGAGTTGGAGGGGAGTGTT-3’

D-CD70-F: 5’-CTGAATCACACAGGACAGCG-3’
D-CD70-R: 5’-CAGTTGGCCAGTGTCACTTG-3’

D-CD1A8-F: 5’-ACATACACCATCCGATCCCC-3’
D-CD1A8-R: 5’-GCTCAGGACATCAGAACCCT-3’

D-CD163-F: 5’-CTTCTGGCCTTTCTCATCGC- 3’
D-CD163-R: 5’-AAGTCAGCACGTCCAGATCA-3’

D-CD1A6-F: 5’-AGCCAGTCTCCTTCCAAGTC- 3’
D-CD1A6-R: 5’-TTCCCGTTCAATCAGCTCCT-3’

D-CD101-F: 5’-CTGTGAGGCAACTGAGTGGA-3’
D-CD101-R: 5’-TCCCAGCCACCACCTACTAC-3’

D-CD93-F: 5’-GCTTTGCCTAGAATCTGCCC- 3’
D-CD93-R: 5’-AAAAGGACAGCTTGCCCATG-3’

D-CD2-F: 5’-GGGCCTGGACATCTATCTCA-3’
D-CD2-R: 5’-GTTGGGACACAGCTGGATTT-3’

D-CD36-F: 5’-TGACCGTTCTCAATCTGGCT- 3’
D-CD36-R: 5’-AGAATGGATCCGTGTAGCCC-3’

D-CD74-F: 5’-GTGGCCTTCTGTGGACAAAT-3’
D-CD74-R: 5’-GCGCTGGTCTTCCATAGTTC-3’
